# An Isotonic Drink Containing Pacific Cod (*Gadus macrocephalus*) Processing Waste Collagen Hydrolysate for Bone and Cartilage Health

**DOI:** 10.3390/md22050202

**Published:** 2024-04-27

**Authors:** Nikita Yu. Zarubin, Elena N. Kharenko, Olga V. Bredikhina, Elizaveta V. Lavrukhina, Kira S. Rysakova, Vitaly Yu. Novikov, Georgy E. Leonov, Igor V. Vakhrushev, Konstantin V. Zolotarev, Anton N. Mikhailov, Marina V. Mikhailova

**Affiliations:** 1 Russian Federal Research Institute of Fisheries and Oceanography, 19 Okruzhnoy Proyezd, 105187 Moscow, Russia; zar.nickita@yandex.ru (N.Y.Z.); harenko@vniro.ru (E.N.K.); bredihinaov@rambler.ru (O.V.B.); chitosan@vniro.ru (E.V.L.); rysakova@pinro.vniro.ru (K.S.R.); nowitaly@yandex.ru (V.Y.N.); 2 Polar Branch, Russian Federal Research Institute of Fisheries and Oceanography, 6 Akademik Knipovich Str., 183038 Murmansk, Russia; 3 Institute of Biomedical Chemistry, 10 Pogodinskaya Str., 119121 Moscow, Russia; golerus@gmail.com (G.E.L.); vakhrunya@gmail.com (I.V.V.); myhas84@mail.ru (A.N.M.); m_mikhailova@mail.ru (M.V.M.)

**Keywords:** bone and cartilage health, bioactive substances, Pacific cod, processing waste utilization, collagen hydrolysate, isotonic drink

## Abstract

Malnutrition is one of the major factors of bone and cartilage disorders. Pacific cod (*Gadus macrocephalus*) processing waste is a cheap and highly promising source of bioactive substances, including collagen-derived peptides and amino acids, for bone and cartilage structure stabilization. The addition of these substances to a functional drink is one of the ways to achieve their fast intestinal absorption. Collagen hydrolysate was obtained via enzymatic hydrolysis, ultrafiltration, freeze-drying, and grinding to powder. The lyophilized hydrolysate was a light gray powder with high protein content (>90%), including collagen (about 85% of total protein) and a complete set of essential and non-essential amino acids. The hydrolysate had no observed adverse effect on human mesenchymal stem cell morphology, viability, or proliferation. The hydrolysate was applicable as a protein food supply or a structure-forming food component due to the presence of collagen fiber fragments. An isotonic fitness drink (osmolality 298.1 ± 2.1 mOsm/L) containing hydrolysate and vitamin C as a cofactor in collagen biosynthesis was prepared. The addition of the hydrolysate did not adversely affect its organoleptic parameters. The production of such functional foods and drinks is one of the beneficial ways of fish processing waste utilization.

## 1. Introduction

Malnutrition is one of the major factors of bone disorders. This has been reported for relatively healthy people with anorexia nervosa [1]. The risk of bone disorders is substantial in the cases of malnutrition for patients with maintenance hemodialysis [2], kidney transplant recipients [3], and patients with Parkinson’s disease [4]. Various malnutrition-related cartilage disorders have also been reported, e.g., chronic inflammatory arthritis [5] and enlargement of cartilage [6]. 

Collagen is the main structural protein of bones, cartilage, ligaments, and tendons in the joints. Hydrolyzed collagen, as a source of specific amino acids, can perform a building function and be a material for the formation of new collagen fibrils in the connective tissues of cartilage, thereby restoring them after damage [7]. Short collagen-derived peptides consisting of two or three amino acids are easily absorbed in the intestine [8]. If collagen-derived peptides are consumed, the process of protein digestion in the gastrointestinal tract is facilitated [9], and the level of amino acids entering the cartilage increases; those amino acids are used further in collagen biosynthesis in chondrocytes [10]. From about 25 years of age, collagen biosynthesis slows down, and with age, the ratio of collagen types in tissues changes. Collagen becomes more rigid, which leads to a deterioration in the condition of all collagen-containing tissues in the human body. Therefore, maintaining musculoskeletal system performance is possible with the use of collagen derivatives in a daily dose of 10 g for 1–6 months [11].

Generally, only 30–50% of the fish wet weight is used in the production of fish fillets [12]. Fish processing waste is mostly used for fish meal or fish oil production, but it is a promising source of biologically active substances, including collagen and its derivatives [13]. Pacific cod (*Gadus macrocephalus*) is one of the most captured fish species in Russia [14]. Pacific cod processing waste actually comprises heads (21.5–25.6% of total wish wet weight); skin (5.2–6.0%); tails, swim bladders, spinal bones, clavicles, and pectoral fins (2.8–14.8%); and viscera: roe, milt, and liver (16.3–23.9%) [15]. It has been reported that some peptides derived from Pacific cod bone via hydrolysis induce anti-osteoporosis effects in rats [16]. Pacific cod skin contains type I collagen; its partial hydrolysate enhances the proliferation of fibroblast and osteoblast cells [17]. 

The addition of biologically active substances to functional drinks is one of the ways to achieve their fast intestinal absorption. The drinks may be supplemented with flavoring ingredients and enriched with functional components, including collagen hydrolysate, for connective tissue regeneration [18,19]. Isotonic drinks are of great biomedical interest because they are developed for the normalization of water and electrolyte content in the body and the correction of imbalance of minor nutrients. When developing the formulation of an isotonic drink, most attention is paid to the concentrations of carbohydrates and minerals contributing to the drink’s osmolarity, which should be within 275–300 mOsm/L [20]. Vitamin C is known to be a cofactor in collagen biosynthesis [21]. Therefore, it is reasonable to combine collagen hydrolysate with citrus fruits (lemon, orange, or grapefruit) with substantial vitamin C content (33.4–97.1 mg/100 g) or their juice concentrates [10,22].

Thus, Pacific cod processing waste may potentially be a cheap and highly promising source of bioactive substances for maintaining the sustainability of bone and cartilage health. A vitamin C-containing isotonic drink is a promising basis for the administration of such substances due to the fast intestinal absorption, increased collagen biosynthesis rate, and positive side effects. The aim of this research was to prepare samples of collagen hydrolysate from Pacific cod processing waste and study their chemical composition and the necessary physicochemical properties for the design of bone and cartilage health-improving functional drink formulations, the manufacturing of which could be a method of fish processing waste utilization.

## 2. Results and Discussion

### 2.1. Chemical Composition of Pacific Cod Processing Waste Components

The chemical composition of the waste components is presented in Table 1. The difference between the components in collagen and protein content was not substantial, except for heads with clavicles and muscle cutoffs and tails with tail fins. Most likely, the deficiency in collagen in those components is caused by the presence of collagen-free muscle. The total fat content of the samples is suitable for subsequent freeze-drying; samples rich in lipids are not recommended for freeze-drying due to the low quality of lyophilizate [23].

### 2.2. Quality and Chemical Parameters of Collagen Hydrolysate

Some values of quality and chemical parameters of the lyophilized collagen hydrolysate are summarized in Table 2. The lyophilizate was formed as porous plates with a width of 3–6 mm, light gray color, insipid odor and flavor, and brittle structure. The plates were subsequently ground to powder which had a satisfactory flow rate according to the value of angle of repose. The powder was partly soluble, but the insoluble fraction was negligible for the purpose of functional drink preparation. The powder had no enzymatic activity and a lightly acidic nature. The quality parameters of the hydrolysate make it acceptable as a food or drink component [24]. 

The molecular weight distribution of the collagen hydrolysate being studied was typical for fish collagen enzymatic hydrolysate [13,25]. The high content of amino acids makes the hydrolysate applicable as a protein food supply and a structure-forming food component due to the presence of collagen fiber fragments. The collagen hydrolysate had a relatively high content of glycine, proline, and hydroxyproline (about 50% in total), which is typical for untreated fish collagen [26] (see Table 3). Trace contents of tryptophan were mostly derived from the fish muscle protein of muscle cutoffs [27,28]. The hydrolysate contained a complete set of essential and non-essential amino acids and is applicable as a protein supply. A low lipid content (<1%) makes the hydrolysate applicable as a component of food or drinks for low-calorie and very low-calorie diets [29]. 

### 2.3. Cell Morphology, Proliferation, and Viability Assay

**Figure 1:**
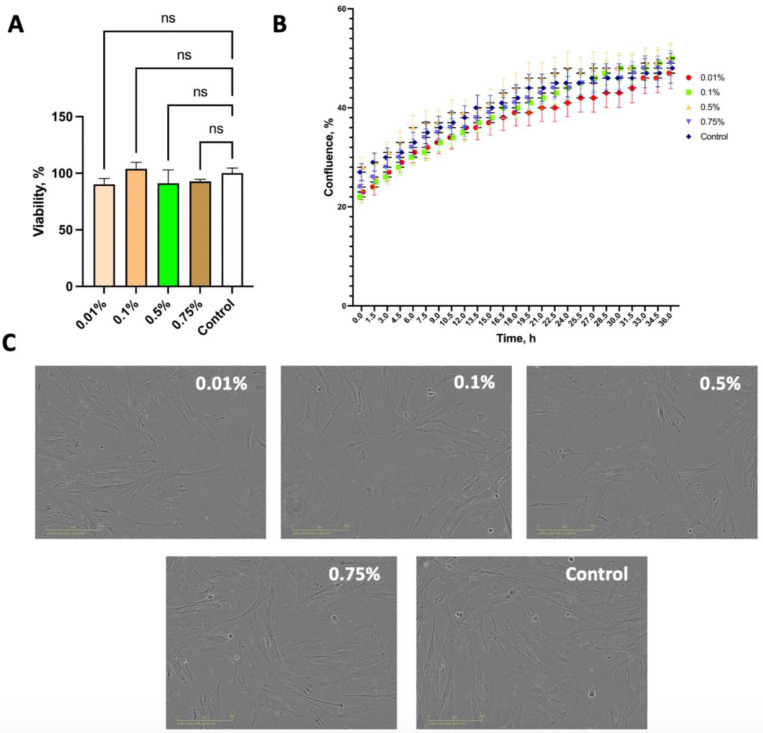
Cell morphology, proliferation, and viability assay for collagen hydrolysate samples (*n* = 3) using human umbilical cord Wharton’s Jelly mesenchymal stem cells (hWJMSCs): (**A**) cell viability after 36 h of incubation with different concentrations of samples, as measured by MTT assay (means ± SD; *n* = 3); ns = not significant (Mann–Whitney *U* test); (**B**) evaluation of the effect of the samples on the proliferation of hWJMSCs by measuring confluence at 1.5 h intervals using real-time imaging (means ± SD; *n* = 3); (**C**) cell morphology during incubation with different concentrations of samples, phase contrast microscopy.

No changes were found in cell morphology 36 h after collagen hydrolysate addition at any of the concentrations used (Figure 1C). The confluence indices also had no significant differences between untreated (control) cells and cells treated with different collagen concentrations (Figure 1B). A 36 h exposure to collagen hydrolysate did not significantly affect cell viability compared with control cells (Figure 1A). The mean values of the viability percentages for cells exposed to 0.01%, 0.1%, 0.5%, and 0.75% collagen hydrolysate were 90.1%, 103.8%, 91.2%, and 91.8%, respectively (Figure 1A). Overall, the results indicate that collagen hydrolysate had no negative effects on human umbilical cord Wharton’s Jelly mesenchymal stem cell (hWJMSC) morphology, viability, or proliferation. 

### 2.4. Isotonic Drink Properties

The applicability of the Pacific cod processing waste collagen hydrolysate as a component of isotonic fitness fruit drinks was studied. The drink formulations (see Section 3.16) were designed to make them isotonic (osmolarity 275–300 mOsm/L). The mineral electrolytes and low-molecular carbohydrates should be included in a fitness drink; their content should be adjusted to achieve the necessary osmolarity level. The addition of wholesome biologically active substances (e.g., proteins, amino acids, and vitamins) is acceptable [20]. In the case of our collagen hydrolysate-containing drink, the necessary osmolarity was achieved by adjusting the concentrations of salt, carbohydrates, and juice concentrates. Citrus fruit juices are also sources of vitamin C, a known cofactor in collagen biosynthesis [21]. Collagen hydrolysate was added to the drink as another bioactive component for bone and cartilage structure stabilization. As mentioned above, it contains a substantial content of proline and hydroxyproline. These two amino acids are crucial for collagen triple helix stability due to the formation of extra hydrogen bonds [30,31]. Proline and hydroxyproline are both non-essential (or conditionally essential) amino acids, but their biosynthesis rate may not cover the requirement of collagen biosynthesis; therefore, they should be additionally consumed for maintaining the sustainability of bone and cartilage health [32].

Some physicochemical and organoleptic parameters of the drink prepared in three flavor variations according to the designed formulations are presented in Table 4. The drink was slightly acidic, which is typical for citrus fruit drinks. The content of hydroxyproline and total collagen-derived amino acids characterizes the drink as an additional source of bioactive substances for collagen biosynthesis, and so does the content of vitamin C; the administration of 500 mL of the drink (in any variation) covers >40% of the recommended daily dose of vitamin C in the United States and Canada [33]. The organoleptic estimation of the drink showed that its odor, flavor, and color were typical for any drink of the respective flavor variation (lemon, orange, or grapefruit) without any influence of the collagen hydrolysate. Despite the partial water solubility of the collagen hydrolysate, the finished drink had no solid matter visible to the naked eye. 

## 3. Materials and Methods 

### 3.1. Materials

Samples of adult Pacific cod (*Gadus macrocephalus*) were obtained from local fish dealers in the Khabarovsk region, Russia. The fish were caught by fishing companies in the northern part of the Sea of Okhotsk. The samples were dissected, and the tissues were mixed in a weight ratio typical for the cod processing waste [34]: heads with clavicles and muscle cutoffs—35%; skin with scales—22%; tails with tail fins—5%; spinal bones—20%; viscera (without roe, milt, or liver)—18%. The mixture samples were washed with water at 8–12 °C for 20 min, kept frozen at −25 °C for 2 h, and ground in a meat grinder with a 3 mm hole grid. Next, the mixture samples were hydrolyzed using *Bacillus licheniformis* protease with 50,000 U/g proteolytic activity (“Protozyme”, manufactured by Biopreparat, Moscow, Russia). Fifty grams of tissue homogenate was mixed with 0.1 g of the enzyme and 50 mL of distilled water. The hydrolysis was performed in a thermostatic water bath at 30 °C for 6 h. Next, the enzyme was inactivated by heating the mixture at 70 °C for 15 min, and the hydrolysate was separated from sediment by filtration through Whatman No. 3 filter paper. The hydrolysate was mixed with 10% citric acid (ratio: 1:1) and treated for 4 h with constant stirring for odor neutralization. Next, the mixture was filtered in a UF-401/402 ultrafiltration system (BioTechno Group, Moscow, Russia) using an aromatic polysulfonamide membrane filter with a 100 kDa nominal molecular weight limit (Vladipor, Vladimir, Russia) at 50 °C. The filtrate was lyophilized in a Lyopro-2.5 freeze dryer (Lyomac, Shanghai, China) in a plastic tray: freezing at −35 °C for 24 h and drying at 30 °C for 5 h. The lyophilizate was finely ground in a mortar. The whole procedure of hydrolysate preparation was performed in triplicate.

The ordinary chemicals used in this study (citric acid, NaOH, HCl, CuSO_4_, H_2_SO_4_, H_2_O_2_, KH_2_PO_4_, p-dimethylaminobenzaldehyde, n-propanol, HNO_3_, K_4_[Fe(CN)_6_], Zn(CH_3_COO)_2_, CH_3_COOH, NH_4_Fe(SO_4_)_2_·12H_2_O, KSCN, AgNO_3_, LiClO_4_, dimethyl sulfoxide (DMSO), formazan, and acetonitrile) were manufactured by Component-Reaktiv, Moscow, Russia, and had high purity or were analytical grade. The chemicals used as standards (sodium caseinate, tyrosine, amino acid standards, and carbohydrate standards) and 3-(4,5-dimethylthiazol-2-yl)-2,5-diphenyltetrazolium bromide (MTT) used in the cell viability assay were manufactured by Sigma-Aldrich, St. Louis, MO, USA, and were analytical grade. The components used for isotonic drink preparation were food quality grade (see Section 3.16).

### 3.2. Water Content Determination

A sample was finely homogenized manually (with scissors and/or mortar). The water content was measured with an MF-50 moisture analyzer (A&D, Tokyo, Japan). Each measurement was made in triplicate. 

### 3.3. Total Protein Content and Total Nitrogen Content Determination

The total protein content was determined as 6.25 × total N content; the latter was determined using the Kjeldahl method with a Kjeltec 1002 System Distilling Unit (Tecator, Höganäs, Sweden). Each measurement was made in triplicate. 

### 3.4. Collagen Content or Total Content of Collagen-Derived Amino Acid Determination

Collagen content, or the total content of collagen-derived amino acids (for collagen hydrolysate and drink samples), was determined as 7.4 × hydroxyproline content [13]. For hydroxyproline content determination, a homogenized sample (about 50 mg) was hydrolyzed by autoclaving in 1.0 mL of 6 M HCl in a sealed tube at a pressure of 3.5 bar for 3 h. Then, 1 mL of 0.01 M CuSO_4_, 1 mL of 2.5 M NaOH, and 1 mL of 6% H_2_O_2_ were added to the sample tube and a blank tube with 1 mL of distilled water. The solutions were mixed with occasional shaking for 5 min each time and later placed in a water bath at 80 °C for 5 min with frequent intensive shaking. Next, the tubes were cooled in ice water, and 4 mL of 3.0 N (1.5 M) H_2_SO_4_ was added with shaking. Then, 2 mL of p-dimethylaminobenzaldehyde solution in n-propanol was added with thorough shaking. The tubes were heated at 70 °C for 16 min and cooled in tap water [35]. The absorbance at 540 nm of the prepared solutions was measured with a PE-5300VI spectrophotometer (Ecroskhim, St. Petersburg, Russia). The hydroxyproline content was calculated using a previously made calibration with the hydroxyproline standard (Sigma-Aldrich, St. Louis, MO, USA). Each measurement was made in triplicate.

### 3.5. Total Fat Content Determination

A sample was finely homogenized manually (with scissors and/or mortar). Total fat was extracted using the SER 148 automated extraction system (VELP Scientifica, Usmate Velate, Italy). The total fat content was measured gravimetrically by weighing the extract. Each measurement was made in triplicate. 

### 3.6. Ash Content Determination

A sample (about 5 g) was digested in a muffle furnace at 500–700 °C for about 1 h to constant weight; the ash was collected and weighed. Each measurement was made in triplicate. 

### 3.7. Powder Flow Determination

Powder flow was determined by measuring the time during which a weighed portion of the hydrolysate passed (flowed) through a funnel using a standardized method accepted in Russian pharmacopoeia [36]. A standardized funnel made of stainless steel with a 110 mm upper inner diameter, a 10 mm lower inner diameter, and a 40° vertical angle was used. A portion that would fill up 80–90% of the funnel was gained and weighed. The lower hole was plugged, the hydrolysate portion was poured through the upper hole, the lower hole was then unplugged, the time of portion passing was measured, and the powder flow was calculated in grams per second as portion weight/passing time. Each measurement was made in triplicate. 

### 3.8. Angle of Repose Determination

The angle of repose was determined as the angle of dip of the pile of hydrolysate formed after passing the funnel during powder flow determination (see Section 3.7) relative to the horizontal plane. The angle was measured with a goniometer from three sides of the pile; the result was averaged. The powder flow rate was estimated using the standardized scale (Table 5). Each measurement was made in triplicate.

### 3.9. Enzymatic Activity Determination

The method is described in detail in [26]. Briefly, the substrate solution of sodium caseinate standard (Sigma-Aldrich, St. Louis, MO, USA) was prepared by mixing 8 mL of 1 M NaOH, 36 g of urea, 10 mL of pre-prepared 22% sodium caseinate solution, and 72 mL of distilled water; keeping that mixture at 25 °C for 30 min; and adding 10 mL of 1 M KH_2_PO_4_ and 4 g of urea. Next, 1 mL of 2.35% hydrolysate suspension (concentration equal to its content in the drink) was added to 5 mL of the substrate solution; the mixture was stirred and kept at 25 °C for 10 min. Then, 10 mL of 0.3 M trichloracetic acid was added, and the mixture was stirred and filtered through Whatman No. 3 filter paper. Subsequently, 10 mL of 0.5 M NaOH was added to 5 mL of the filtrate, and 3 mL of phenol reagent [37] was rapidly added with stirring. The standard solution was prepared by adding 0.145 mg of tyrosine to 5 mL of 0.2 M HCl. Next, 10 mL of 0.5 M NaOH was added to 5 mL of the mixture, and 3 mL of phenol reagent [37] was rapidly added with stirring. After 5 min, enzymatic activity was estimated by measuring tyrosine concentration using a KFK-3-01 colorimeter (Zagorsk Optical-Mechanical Plant, Sergiyev Posad, Russia) with a red filter against the standard solution. Each measurement was made in triplicate. 

### 3.10. Determination of pH

A SevenExcellence pH meter with InLab Expert Go-5m-ISM electrode (Mettler Toledo, Greifensee, Switzerland) was used to measure the pH value of the samples. Each measurement was made in triplicate. 

### 3.11. Molecular Weight Analysis

The molecular weight distribution of 1% hydrolysate suspension filtered through Whatman No. 3 filter paper was analyzed using size-exclusion high-performance liquid chromatography with an LC-10Avp chromatographer with an SPD-10Avp UV/VIS Detector (Shimadzu, Columbia, MD, USA) equipped with TSKgel Alpha-M (30 cm × 7.8 mm, 13 μm particle size) and TSKgel Alpha-2500 (30 cm × 7.8 mm, 7 μm particle size) columns (Tosoh, Tokyo, Japan); 0.15 M NaCl solution was used as eluent with a 0.8 mL/min elution speed. Absorbance was measured at 210 and 280 nm. The analysis was performed in triplicate. 

### 3.12. Amino Acid Analysis

A total of 10 mg of a sample was mixed with 1 mL of HPLC-grade water. Fifty microliters of 25× diluted suspension was dried in an ampule under vacuum. Next, 100 µL of 6 M HCl was added, the ampule was sealed, and hydrolysis was performed at 110 °C for 24 h. Then, the ampoule was unsealed and dried in a vacuum concentrator, and 50 µL of 0.1 M HCl was added. The analysis was performed using an Agilent 1200 series chromatographer with a fluorescent detector and a ZORBAX Eclipse AAA (15 cm × 4.6 mm; 5 µm particle size) column (Agilent, Santa Clara, CA, USA). The mobile phase was prepared from 40 mM pH 7.8 phosphate buffer (Solution 1) and 80% acetonitrile (Solution 2). The amino acids were derivatized by adding 1 mL of derivatizing solution (50 mg of o-phthaldialdehyde was dissolved in 1 mL of methanol, and then 9 mL of 100 mM borate buffer (pH 10.2) was added); the mixture was left for 5 min at room temperature. The derivatives were eluted at a flow rate of 1 mL/min with a gradient of Solution 2; the total run time was 41 min. The concentrations of the amino acids were calculated using the previously made calibration with amino acid standards. Some other details of the procedure are described in [38]. The analysis was performed in triplicate. 

### 3.13. Cell Cultures

Primary cultures of mesenchymal stem cells from human umbilical cord Wharton’s Jelly (hWJMSCs) were cultured in DMEM/F12 (PanEco, Moscow, Russia) supplemented with 10% fetal bovine serum (FBS; Gibco, Carlsbad, CA, USA), 2 mM L-glutamine (PanEco, Moscow, Russia), 100 U/mL penicillin (PanEco, Moscow, Russia), and 100 μg/mL streptomycin (PanEco, Moscow, Russia). HWJMSCs were seeded in 24-well plates at 2 × 10^3^ cells per well and cultured for 12 h in a CO_2_ incubator at 37 °C, 5% CO_2_, and 95% humidity. Following this, collagen hydrolysate samples at concentrations of 0.01%, 0.1%, 0.5%, and 0.75% in Dulbecco’s phosphate-buffered saline (DPBS, Gibco, Carlsbad, CA, USA) were added to cultured cells. Control cells received an equivalent volume of DPBS alone. Cell culture lasted for an additional 36 h after the addition of collagen, and then cell viability and proliferation were evaluated. 

### 3.14. Cell Viability Assay

Cell viability was assessed with an MTT assay. Briefly, 400 μL of MTT solution (5 mg/mL) was added to each well of a 24-well plate, followed by a 2 h incubation. Formazan crystals were then dissolved in DMSO, 100 μL per well, and the absorbance was measured at 570 and 620 nm using an Infinite M200 Pro tablet reader (Tecan, Männedorf, Switzerland). The results were normalized to the absorbance of the control cells, which was assumed to be 100%. The assay was performed in triplicate.

### 3.15. Cell Proliferation Assay

Real-time images of cell confluence were obtained at 1.5 h intervals for 36 h using an IncuCyte ZOOM Live-Cell Imaging System (Sartorius, Göttingen, Germany). A quantitative assessment of cell proliferation was performed by analyzing time-scan curves constructed using the proprietary software of the IncuCyte ZOOM system (Sartorius, Göttingen, Germany). The assay was performed in triplicate.

### 3.16. Isotonic Drink Preparation

The samples of the isotonic drink were prepared according to their formulations presented in Table 6. The fruit juice concentrates were prepared from natural fruits purchased in food stores in Moscow, Russia. The fruits were washed with hot tap water; next, their pulp was separated from the rind, and juice was extracted from the pulp with a manual squeezer and filtered through four layers of gauze. The components were mixed in a Turbula 2.0 laboratory mixer (Vibrotechnik, St. Petersburg, Russia) for 10 min at a 50 min^−1^ rotation speed. The procedure of isotonic drink preparation was performed in triplicate.

### 3.17. Osmolarity Determination

The osmolarity of the drink samples was measured with an Osmo Station OM-6060 automatic freezing point depression osmometer (ARKRAY, Kyoto, Japan). Each measurement was made in triplicate. 

### 3.18. NaCl Content Determination

First, 80 mL of a drink sample was mixed with 1 mL of 0.25 M K_4_[Fe(CN)_6_], and then 1 mL of 1 M Zn(CH_3_COO)_2_ dissolved in 3% CH_3_COOH was added, the mixture was vigorously shaken, and the volume was adjusted to 100 mL with distilled water (Solution 1). 

Five hundred grams of preliminarily ground NH_4_Fe(SO_4_)_2_·12H_2_O was dissolved in 1 L of boiling distilled water. Then, the solution was cooled with cold tap water and filtered through Whatman No. 3 filter paper. Concentrated HNO_3_ was added to the filtrate in small portions until the solution became transparent (Solution 2). 

The titration burette was filled with 0.1 M KSCN. Another titration burette was filled with 0.1 M AgNO_3_. Next, 5 mL of concentrated HNO_3_, 2 mL of Solution 2, and 2 drops of 0.1 M KSCN from the burette were added to Solution 1, and the mixture was shaken. Next, 0.1 M AgNO_3_ was added from the burette with constant shaking until the red color disappeared, with a little excess. Then, 0.1 M KSCN from the first burette was added until stable within 30 s of a red-brown color appearing.

The same procedure was performed with 80 mL of distilled water instead of a drink as a blank test. NaCl content was calculated in g/L as 0.073 × ((volume of 0.1 M AgNO_3_ expended for drink sample (mL) – volume of 0.1 M AgNO_3_ expended for blank sample (mL)) – (volume of 0.1 M KSCN expended for drink sample (mL) – volume of 0.1 M KSCN expended for blank sample (mL))). Each measurement was made in triplicate. 

### 3.19. Total Carbohydrates Content Determination

The content of total carbohydrates was determined using BioLC Dionex high-performance ion chromatographer equipped with a CarboroPac PA 1 (25 cm × 4 mm, 10 μm particle size) column (Thermo Fisher Scientific, Waltham, MA, USA). HPLC grade distilled water was used as eluent with a 1.0 mL/min elution speed. The content of each carbohydrate was measured by the peak area calibrated with solutions of standards of all detected carbohydrates. The content of total carbohydrates was calculated as the sum of the content of all detected carbohydrates. Each measurement was made in triplicate. 

### 3.20. Vitamin C Content Determination

The method is described in detail in [39]. Briefly, vitamin C was determined using a Milichrom A-02 high-performance liquid chromatographer equipped with a Prontosil 120-5 C18 (7.5 cm × 2 mm, 5 μm particle size) column (EcoNova, Novosibirsk, Russia). A gradient of 0.4 M LiClO_4_ (pH 2.4) and acetonitrile was used as the mobile phase. Vitamin C was detected at a 2.5 min retention time and quantified by measuring the absorbance at 240 nm. Each measurement was made in triplicate. 

### 3.21. Viscosity Determination

The viscosity of the drink samples was measured with a Polymer RPE-1M rotational viscosimeter equipped with a T1-B1 cylinder system (Khimpribor-1, Tula, Russia). Each measurement was made in triplicate. 

### 3.22. Statistical Analysis

The numerical data are presented as means ± SD. The significance of the difference between the groups of samples being compared (Table 1, Figure 1) was evaluated using the Mann–Whitney *U* test; the level of significance considered was *p* < 0.05. Data analysis was performed using STATISTICA 9.0 software.

## 4. Conclusions

Pacific cod processing waste is a mass-production waste; therefore, it is a promising source of low-cost collagen and other bioactive substances. The hydrolysate of collagen obtained from Pacific cod processing waste is rich in amino acids, especially proline and hydroxyproline, which are necessary for collagen biosynthesis. The hydrolysate had no observed adverse effect on hWJMSC morphology, viability, or proliferation. The quality parameters and negligible cytotoxicity of the hydrolysate make it acceptable as a protein or structure-forming component of food or drinks, including food or drinks for low-calorie and very low-calorie diets. The addition of protein hydrolysate to a functional drink is one of the ways to achieve fast intestinal absorption of its components. The hydrolysate-containing isotonic fitness drink had acceptable physicochemical and organoleptic parameters and a substantial content of vitamin C, a known cofactor in collagen biosynthesis. Therefore, the designed functional drink is a promising source of bioactive substances beneficial for the sustainability of bone and cartilage health. The mass manufacturing of such drinks is one of the beneficial ways of fish processing waste utilization.

## Figures and Tables

**Table 1:** Chemical composition of Pacific cod processing waste components.

Component of Waste	Water Content	Total Protein Content	Collagen Content	Total Fat Content	Ash Content
Heads with clavicles and muscle cutoffs	78.21 ± 1.43 ^a^	15.71 ± 0.39 ^a^	10.02 ± 0.24 ^a^	0.77 ± 0.01 ^a^	5.37 ± 0.14 ^a^
Skin with scales	75.79 ± 1.39 ^b^	18.61 ± 0.46 ^b^	14.38 ± 0.34 ^b^	1.03 ± 0.02 ^b^	3.56 ± 0.08 ^b^
Tails with tail fins	79.55 ± 1.45 ^a^	14.38 ± 0.36 ^a^	11.52 ± 0.27 ^a^	0.53 ± 0.01 ^c^	5.54 ± 0.14 ^a^
Spinal bones	74.32 ± 1.36 ^b^	18.25 ± 0.45 ^b^	13.51 ± 0.32 ^b^	0.75 ± 0.01 ^a^	6.68 ± 0.15 ^c^
Viscera (without roe, milt, or liver)	74.47 ± 1.36 ^b^	19.96 ± 1.36 ^b^	14.98 ± 0.35 ^b^	2.58 ± 0.06 ^d^	2.99 ± 0.06 ^b^

Values are expressed as % by wet weight (means ± SD; *n* = 3). Different superscripts in each column indicate significant differences (Mann–Whitney *U* test: *p* < 0.05).

**Table 2:** Quality and chemical parameters of lyophilized collagen hydrolysate.

Parameter	Value
Appearance before grinding	Porous plates
Appearance after grinding	Homogenous finely dispersed powder
Odor	Insipid
Flavor	Insipid
Color	Light gray
Powder flow (g/s)	0.87 ± 0.05
Angle of repose (°)	43.5 ± 2.1
Solubility in water	Partial
Enzymatic activity	None
pH of 10% water suspension	4.7 ± 0.2
Molecular weight (kDa)	60.7 ± 49.7
Water content (% by weight)	5.4 ± 1.2
Total protein content (% by weight)	92.1 ± 1.6
Total content of collagen-derived amino acids (% by total content of amino acids)	85.2 ± 1.1
Total fat content (% by weight)	0.8 ± 0.2
Ash content (% by weight)	1.9 ± 0.2

Numerical values are expressed as means ± SD; *n* = 3.

**Table 3:** Amino acid composition of lyophilized collagen hydrolysate.

Amino Acid	Content (% by Weight)
Lysine	4.23 ± 0.16
Histidine	1.49 ± 0.04
Arginine	4.58 ± 0.1
Aspartic acid	5.7 ± 0.13
Threonine	2.33 ± 0.05
Serine	2.88 ± 0.06
Glutamic acid	10.09 ± 0.16
Proline	9.32 ± 0.17
Hydroxyproline	7.84 ± 0.18
Tryptophan	0.11 ± 0.01
Cysteine	0.13 ± 0.01
Glycine	28.51 ± 0.61
Alanine	5.05 ± 0.11
Valine	2.1 ± 0.05
Methionine	0.05 ± 0.01
Isoleucine	2.27 ± 0.05
Leucine	3.26 ± 0.07
Tyrosine	0.04 ± 0.01
Phenylalanine	1.3 ± 0.03
Hydroxylysine	0.79 ± 0.02

Content values are expressed as means ± SD; *n* = 3.

**Table 4:** Physicochemical and organoleptic parameters of isotonic drink containing lyophilized collagen hydrolysate.

Parameter	Lemon Drink	Orange Drink	Grapefruit Drink
Osmolarity (mOsm/L)	299.1 ± 1.1	297.5 ± 1.6	298.3 ± 1.8
NaCl content (g/L)	5.7 ± 0.4 ^a^	6.1 ± 0.3 ^b^	5.8 ± 0.4 ^a^
Total carbohydrate content (g/L)	46.5 ± 3.6	42.3 ± 3.4	44.1 ± 2.3
Total nitrogen content (g/L)	3.9 ± 0.3	3.3 ± 0.2	3.6 ± 0.3
Total collagen-derived amino acids content (g/L)	19.9 ± 1.2	18.0 ± 1.0	20.5 ± 1.4
Vitamin C content (mg/L)	75.5 ± 8.9 ^a^	90.4 ± 10.4 ^b^	72.4 ± 9.3 ^a^
pH	4.5 ± 0.1	4.8 ± 0.1	4.6 ± 0.0
Viscosity (kPa∙s)	1.14 ± 0.23	1.16 ± 0.19	1.19 ± 0.24
Color	Light yellow	Yellow	Light red
Odor	Lemon-like	Orange-like	Grapefruit-like
Flavor	Sour-sweet	Sour-sweet	Sour-sweet
Transparency	Homogeneously turbid	Homogeneously turbid	Homogeneously turbid

Numerical values are expressed as means ± SD; *n* = 3. Different superscripts in each row indicate significant differences between numerical values (Mann–Whitney *U* test: *p* < 0.05).

**Table 5:** Scale for powder flow rate estimation by angle of repose [36].

Powder Flow Rate	Angle of Repose (°)
Very good	<30
Good	30–35
Satisfactory	35–45
Unsatisfactory	45–55
Bad	55–65
Very bad	>65

**Table 6:** Isotonic drink formulations. The purchased components were food quality grade.

Component	Manufacturer	Content (% by Weight)		
		Lemon Drink	Orange Drink	Grapefruit Drink
Drinking water	Svyatoy Istochnik, Kostroma, Russia	75.14	78.89	76.07
Fruit juice concentrate	Authors; extracted from natural fruits	18.78	15.03	17.85
Fructose	Molecularmeal, Moscow, Russia	2.09	2.09	2.09
Glucose	Molecularmeal, Moscow, Russia	1.17	1.17	1.17
Collagen hydrolysate	Authors	2.35	2.35	2.35
Sea salt	Mareman, Tashkent, Uzbekistan	0.47	0.47	0.47

## Data Availability

The original data presented in the study are included in the article; further inquiries can be directed to the corresponding author.
